# Retrospective observational evaluation of postoperative oxygen saturation levels and associated postoperative respiratory complications and hospital resource utilization

**DOI:** 10.1371/journal.pone.0175408

**Published:** 2017-05-17

**Authors:** Satya Krishna Ramachandran, Aleda Thompson, Jaideep J. Pandit, Scott Devine, Amy M. Shanks

**Affiliations:** 1 Department of Anesthesia, Critical Care, and Pain Medicine, Beth Israel Deaconess Medical Center and Harvard Medical School, Boston, Massachusetts, United States of America; 2 Department of Anesthesiology, University of Michigan, Ann Arbor, Michigan, United States of America; 3 Nuffield Department of Anaesthesia, Oxford, Oxfordshire, United Kingdom; 4 Center for Observational & Real-world Evidence: US Evidence & Value Strategies, Merck, Sharpe and Dohme, Whitehouse Station, New Jersey, United States of America; Cleveland Clinic Lerner College of Medicine of Case Western Reserve University, UNITED STATES

## Abstract

**Introduction:**

The clinical importance of postoperative episodic hypoxemia is still unclear, and therefore largely under-studied. As a result, there is limited understanding of its relationship with early postoperative respiratory complications (PRC, defined as intubation within three days of surgery) and hospital resource utilization.

**Materials and methods:**

This single center study was performed using a retrospective observational design. We described population based definitions of desaturation from continuous SpO_2_ monitoring data captured in the post anesthesia care unit (PACU), namely median SpO_2_ in PACU, duration of desaturation below median, nadir desaturation, and length of oxygen therapy relative to PACU duration. These measures were evaluated against the occurrence of early PRC in logistic regression models. Measures that were independently associated with early PRC were accepted as the primary study exposures. Stratified logistic regression models were planned if significant interaction occurred with high risk surgical procedures. Models were adjusted by including several patient conditions, procedural, and anesthesia risk factors. Propensity matching on desaturation occurrence was planned to evaluate the relationship with postoperative resource utilization.

**Results:**

Among 125,740 patients included in the univariate analyses, 351 patients (0.3%) developed early PRC. Nadir desaturation <89% [14.3% of patients; adjusted odds ratio 2.02; 95% CI 1.52, 2.68; p<0.001] and PACU oxygen therapy requirements greater than 60 min [adjusted odds ratio 1.92 (>60 min) to 3.04 (>90 min); p<0.001] were identified as independent predictors of early PRC occurrence. A modest interaction was observed between desaturation and higher surgical risk. Propensity matching for postoperative oxygen requirement was performed in 37,354 matched patients. Matched analysis demonstrated significant increase in day of surgery charges, respiratory charges, total charges, hospital length of stay, reintubation and use of invasive or non-invasive ventilatory support.

**Conclusions:**

In summary, we report that prolonged PACU oxygen therapy and nadir desaturation <89% in PACU as captured in a retrospective database are independently associated with early PRC. This study describes resource implications of PACU desaturation in a large academic medical center in North America.

## Introduction

Early postoperative respiratory complications (PRC) are associated with significant cost and mortality implications for patients.[1,2] Postoperative episodic hypoxemia is understudied in surgical patients, but may have significant value in predicting the risk of early postoperative respiratory complications (PRC). In non-surgical patients, episodic desaturation is associated with a significant increase in major complications. Severe hypoxemia is associated with higher risk of ventricular arrhythmia in patients with obstructive sleep apnea (OSA).[3,4] Episodic hypoxemia is associated with worse outcomes following acute medical emergencies such as myocardial infarction. Sudden death associated with OSA occurs predominantly during sleep, and is associated with more severe nocturnal desaturation and apnea severity.[5,6] The relationship between hypoxemia and respiratory morbidity in postoperative period is perhaps best studied in patients with OSA. Chung et al showed that more subtle desaturation episodes associated with breathing abnormalities were more frequent on the first night after surgery but progressively worsened 3–5 days postoperatively, a finding that has been described over the last two decades.[7–10] In contrast, the risk of unanticipated early postoperative respiratory failure is greatest in the first 24 hours after surgery.[1,11–13]

Perhaps more interestingly, the incidence of significant desaturation in the post-anesthesia care unit (PACU) is significantly greater (16.9–43.8%) than the frequency of early PRC (0.2–3.8%).[14,1] Possibly related to this signal-to-noise ratio, the use of continuous pulse oximetry has been questioned in the past.[15,16] Indeed, the role of early postoperative desaturation in predicting greater risk of PRC has not been explored using a large database with adequate adjustment of other risk factors of interest. This is partly because perioperative measures of significant desaturation are poorly defined and evaluated, despite the fact that they carry significant clinical and legal implications. Most measures relate to single occurrences of threshold desaturation, without dimensions of duration or frequency. More accurate composite measures such as area-under-the-threshold that include both duration and severity are clinically cumbersome. Additional challenges of using retrospective SpO_2_ data in highly monitored environments are that they tend to be addressed either by patient arousals secondary to alarm noise, or by nurse intervention. These interventions range from stimulating the patient to increasing the inspired oxygen fraction. So, it was anticipated that the majority of SpO2 values (>95%) in the PACU would fall in the normal range. At the outset of this study, we assumed that patients manifest PACU desaturation and are managed in one or more ways, necessitating evaluation of more than one measure of desaturation. We sought to derive population thresholds that described dimensions of depth, duration, and clinical response to PACU desaturation from a large database of postoperative patients.

The economic implications of postoperative episodic hypoxemia are also unclear, as they too are largely under-studied. This study aims to address this knowledge gap by describing the association between oxygen desaturation measures and early PRC in a cohort of inpatient post-operative patients. Additionally, we also set out to evaluate the impact of desaturation events on resource utilization measured as charge data and length of stay.

## Materials and methods

This study was performed on the anesthesia information management system database at the University of Michigan, using a retrospective observational design. Data between January 1^st^ 2007 and June 2^nd^ 2014 relating to patient comorbidity, anesthesia techniques, surgical procedure and early postoperative pulse oximetry monitoring of adult patients (18 years and older) were extracted for the study. Institutional Review Board (IRB# HUM00095480) approved the study with a waiver of need for patient consent. Data sources for the study included Centricity Anesthesia Information Management System (GE Healthcare, Waukesha, USA) and the local multicenter perioperative outcome group data as described in several previous studies.[17–20] All data were evaluated for completeness, and text entries were manually coded using a de-identified interface provided by the multicenter perioperative outcome group system.[20]

### Patient population

Study exclusions were as follows: preoperative documentation of existing airway (i.e., endotracheal tube or tracheostomy in situ), preoperative ventilator dependence, postoperative direct ICU admission bypassing PACU care, pediatric age group (age younger than 18 years), and lack of PACU record.

### Study exposures

The primary exposure in the study was postoperative desaturation. In the PACU database, SpO_2_ data samples are captured at every minute during the PACU stay. The validated measures of oxygen desaturation, as seen with the definition of apnea-hypopnea index, are described for higher frequency SpO_2_ data. For instance, scoring of respiratory events in OSA typically uses SpO_2_ data sampled at one second.[21] Perioperative measures of significant desaturation are poorly defined and evaluated, with significant clinical and legal implications. Most measures relate to single occurrences of threshold desaturation, without dimensions of duration or frequency.[22] Additional challenges of using retrospective SpO_2_ data in highly monitored environments are that they tend to be addressed either by patient arousals secondary to alarm noise, or by nurse intervention. These interventions range from stimulating the patient to increasing the inspired oxygen fraction.[23] So, it was anticipated that the majority of SpO_2_ values (>95%) would fall in the normal range. In order to maximize the discriminative ability of a threshold measure, we examined the central tendency measures and spread of SpO_2_ data. Accordingly, we developed metrics for describing early postoperative desaturation across the dimensions of duration, central tendency and nadir levels of desaturation along with the requirement for supplemental oxygen. We planned to evaluate these measures in multiple sensitivity analyses and identify the best measure for outcome prediction. All study patients had a minimum of 120 minutes of SpO_2_ data in PACU extracted for analysis. SpO_2_ data were censored at the first 120 minutes in patients who stayed for longer durations.

#### Central tendency measure

The median SpO_2_ value for each sliding window (5 minute window, q1-minute) was first calculated. On a per patient basis, the median of all sliding window medians was defined as the median SpO_2_ value in PACU. All median data were transformed into a dichotomous measure of central tendency of desaturation by determining the population spread of these data, and identifying the 10^th^ centile of desaturation medians, since lower median values likely signify greater respiratory risk. For this dataset, the median SpO_2_ threshold was defined as <94% using this methodology.

#### Duration of desaturation

The cumulative time in minutes below median SpO_2_, expressed as number of minutes per hour of PACU SpO_2_ monitoring was calculated. All duration data were transformed into a dichotomous measure of duration of desaturation by determining the population spread of these data, and identifying the 90^th^ centile of desaturation duration, since longer desaturation periods likely signify greater respiratory risk. For this dataset, the desaturation duration threshold was defined as the cumulative time ≥18 minutes per hour of SpO2 <94% using this methodology.

#### Nadir desaturation measure

The minimum SpO_2_ in PACU was chosen as the lowest value either documented as a manual entry by PACU nurse during room air exposure for five minutes or captured during all continuous monitoring periods starting from the first recording of SpO_2_ through to PACU discharge. All nadir desaturation data were transformed into a dichotomous measure of nadir desaturation by determining the population spread of these data, and identifying the 10^th^ centile of each patient’s desaturation nadirs, since lower nadir values likely signify greater respiratory risk. For this dataset, the nadir SpO_2_ threshold was derived as <89% using this methodology.

#### Duration of oxygen therapy in PACU

As PACU nurses were expected to treat desaturation with patient arousal and supplemental oxygen therapy, we assumed that longer oxygen exposure times were at least in part, indicative of ongoing desaturation issues needing this intervention. This variable was derived as the fraction of PACU time that the patient had documented oxygen therapy. The derived fraction was converted into an ordinal variable with <25%, 25–49%, 50–74% and 75–100% forming the categories that describe longer oxygen requirements in PACU. For this dataset, these durations were derived as <30 min, 31–60 min, 61–90 min and >90 min using this methodology.

### Study outcomes

The primary health outcome of interest for this study was early PRC defined as postoperative reintubation or intubation of a previously non-intubated patient within the first three days after anesthesia end.[1] As described previously, the study outcome was identified through multiple sources: clinical documentation AIMS system by anesthesia or nursing staff, free text search for “intubation” within the intraoperative and PACU charts, safety event reporting of emergent intubation, documentation of laryngoscopy after tracheal extubation in the operating room or PACU, and documentation of postoperative intubation using preexisting procedure notes.

The secondary outcome of this study was resource utilization as measured by total charges of inpatient care, total charges of care on day of surgery, respiratory therapy charges, pre-surgical charges, pure surgical charges, and hospital-length-of-stay. This analysis was intended to describe healthcare resource use and costs of post-operative oxygen desaturation due to any underlying cause.

### Statistical analysis

Descriptive statistics were generated for all continuous (mean, median, interquartile range, min, max) and categorical (percentage of each response) variables. Where relevant, the extent of missing data was determined. If a variable has more than 5% of values missing, it was evaluated for exclusion. Univariate descriptive statistics were individually performed on variables of interest to determine associations with early PRC. Patient level variables included alcohol intake, smoking, preoperative opioid or benzodiazepine intake, preoperative steroid use, myocardial infarction, congestive heart failure, chronic obstructive pulmonary disease, preoperative pneumonia, pulmonary hypertension, liver disease, renal disease, neurological conditions, seizures, coma, cancer, sepsis, American Society of Anesthesiologists physical status and admission type (outpatient, admission on the day of surgery or inpatient). Intraoperative anesthesia interventions evaluated were anesthesia technique (regional analgesia, general anesthesia or combined), anesthetic agents (propofol infusion, volatile anesthesia, volatile with propofol infusion), general anesthetic exposure (population quartiles of the time duration of estimated minimum alveolar concentration [MAC] above 1.0),[24] number of packed red blood cell units and fresh frozen plasma units transfused, intraoperative opioid agents used, number of intraoperative opioids used, dose of intraoperative opioids (population quartiles of intraoperative morphine equivalent dose normalized for ideal body weight and anesthesia time), use of naloxone, and aminoglycoside administration. We used a composite approach to isolate the NMBA exposures of interest. We first limited the number of NMBAs of interest to three drugs: vecuronium, cistracurium and rocuronium in order to account for the fact that these agents are predominantly used in current state. Next, we normalized the NMBA dose by adjusting for ideal body weight, and anesthesia time. We identified the population distribution of each of these intraoperative NMBA dose per kg per minute and converted them into a binary concepts of high dose (equal to or greater than 75th centile) for each NMBA separately. For this dataset, these were defined as a dose greater than in mg per kg body weight per hour 0.115 mg.kg^-1^.hr^-1^ for cisatracurium, 0.069 mg.kg^-1^.hr^-1^ of vecuronium and 0.494 mg.kg^-1^.hr^-1^ of rocuronium. The relationship between NMBA exposures and outcomes was evaluated by inclusion of these independent categorical variables in the logistic regression models: NMBA used (none, cisatracurium, rocuronium or vecuronium), use of high dose of NMBA, use of neostigmine reversal and use of neuromuscular monitoring. During the study period, sugammadex was not used clinically in the institution. The surgical complexity score was derived using a previously established technique from the primary CPT code.[25,26] This approach computes a continuous score using a logistic regression model to predict the study primary outcome. The continuous surgical complexity score was converted to a dichotomous variable as higher risk (≥75^th^ centile score) or lower risk (<75^th^ centile score), to simplify surgical risk exposure. All patient and operative characteristics were compared using the Student t test or Mann–Whitney U test for continuous variables and the Pearson chi-square test for categorical variables.

#### Validation of desaturation measures

In order to understand the impact of various definitions of the desaturation measures, we developed multivariate logistic regression models to determine the relationship between each measure of desaturation and PRC, after adjusting for previously described risk factors for the study outcome or identified to have univariate associations with the outcome. Desaturation categories were considered the primary independent variable in each model. Desaturation measures independently associated with early PRC (p <0.05) were identified as primary exposure variables for the primary study outcome models.

#### Interaction between surgical service and early postoperative desaturation

The influence of surgical risk on the relationship between desaturation measures and early PRC was first evaluated by examining an interaction model with a 2x2 matrix. If there was evidence of significant interaction, subsequent analyses were stratified by higher and lower surgical risk strata.

#### Logistic regression model

Each of the preoperative, and intraoperative characteristics indicated above were evaluated as independent risk factor variables. We evaluated the assumption of linearity of logits for each ordinal or continuous exposure or covariate by plotting the logits and examining the incremental ORs to determine the need for ordinal or indicator variable coding. Variables with a linear relationship with the outcome were used as ordinal categorical variables, whereas variables were coded as indicator variables when the assumption of linearity was not met. Once the form of the variable was finalized, each variable was initially included in the multivariate model. To determine if a variable should remain in the final multivariate model, we applied a change in estimate criteria. Starting with the fully specified model, the covariate with the largest Wald p-value was removed. When the change in estimate as judged by ln[ORfull/ORfull-1] was >10%, the covariate was retained as a potential risk factor. When the change in estimate was <10%, the covariate was removed from the model. To arrive at a parsimonious set of characteristics in the final multivariate model, the assessment continued until the first variable was reached that produced a change in estimate of >10%. Study estimates were presented as Adjusted Odds Ratios (AOR) and 95% confidence intervals (CI). Missing data frequencies were examined for presence of >5% missingness. In this eventuality, we planned to perform unadjusted 2x2 analyses to compare the PRC rates between patients with missing and complete data.

#### Model diagnostics

Model discrimination was examined by the c-statistic. Model diagnostics were performed to test model stability and multicollinearity. Variables with variance inflation factors of greater than 10 were considered suspect, and thus evaluated for removal from the model. Additionally, the Hosmer-Lemeshow test and condition indices were evaluated. A condition index threshold of 30 was imposed, in the eventuality of which we planned Pearson correlation matrices to identify specific pairs of correlated variables. The goodness-of-fit of the models was evaluated using the omnibus tests of model coefficients. If there was poor fit, we planned to perform internal model validation using Somers Dxy optimism estimates on the bootstrapped data.

#### Secondary outcomes

We developed a propensity score model to estimate incremental resource use and costs of post-operative oxygen desaturation. A logistic regression equation was constructed to describe predictors of post-operative oxygen desaturation for use in assembling propensity score matched cohorts of patients with, and without, post-operative oxygen desaturation. Matching from within the non-oxygen desaturated group was conducted without replacement using an algorithm of one-to-one nearest neighbor matching. Variables in the regression equation included patient age, sex, weight, major co-morbidities, ASA physical status, smoking status, surgical procedure, NMBA use, opioid use and any other variables which may differ between patients with, and without, post-operative oxygen desaturation. Variables pertaining only to the post-PACU phase of a patient’s care were not included in the regression equation to avoid over-controlling for factors which may be an outcome rather than a determinant of oxygen desaturation. Endpoints like hospital length of stay, ICU cost and total inpatient cost can be highly dependent on the type of procedure performed. To strengthen the ability of the propensity score approach to generate appropriate matches so as to isolate true differences in these outcomes in association with post-operative oxygen desaturation, we excluded uncommon procedures (e.g., those with fewer than 50 surgeries available in the dataset). We evaluated the ability of the model to accept the specific surgical procedure as a variable in the regression equation. This was achieved by introducing the CPT codes for specific surgical sites using Raval’s technique.[26] This step was done to overcome a major limitation of prior studies, which only control for high-level procedural category with the potential for a lot of residual unexplained variation. Once propensity score matching was achieved, we performed a test of the strength of the matching by comparing values for variables which would not be expected to differ in a well-matched cohort. These would include the average charges for the surgical procedure itself excluding any post-operative care. Once match balance was achieved, secondary outcomes were compared between the matched groups for continuous outcomes using the Student t test or Mann–Whitney U test. Statistical analysis was performed using PASW^®^ version 20 (SPSS Inc., Chicago, IL).

## Results

**Fig 1:**
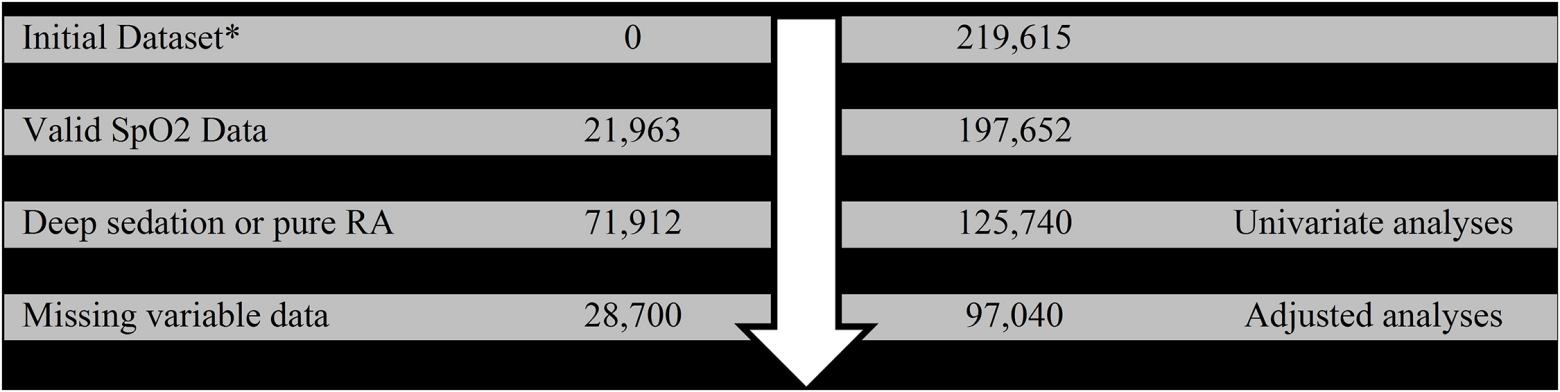
Study flow showing inclusions and exclusions.

After study exclusions, 125,740 patients were included in the univariate analyses (Fig 1). Of these, 351 patients (0.3%) developed early PRC. Measures of significant desaturation were seen in 10.7–22% of patients in the study (Table 1; nadir desaturation values < 89% in 14.3% of patients; median SpO2 <94% in 14.5%; duration >18 min of SpO_2_ <94% in 10.7%; and 21% of each increasing oxygen treatment quartile). All desaturation measures were more frequently observed among patients who developed early PRC.

**Table 1:** Frequency distribution of study variables across outcome groups.

Variable	No early PRC N = 125,389	Early PRC N = 351	p-value	Odds Ratio (95% CI)	% Missing
**PACU oxygen desaturation measures**					
Nadir desaturation <89%	17,895 (16)	111 (37)	<0.001	3.2 (2.5–4.1)	7.9
Median SpO_2_ <94%	18,207 (16)	61 (21)	0.037	1.4 (1.0–1.8)	11.3
Duration >18 min of SpO_2_ <94%	13,335 (13)	73 (26)	<0.001	2.3 (1.8–3.0)	18.2
PACU oxygen treatment duration quartiles			<0.001	N/A	
<30 minutes	27,614 (26)	18 (7)			
31–60 minutes	26,198 (25)	56 (21)			
61–90 minutes	26,278 (25)	92 (35)			
>90 minutes	26,841 (25)	99 (37)			
***Preoperative conditions***					
**Risk for OSA (PSAP categories)**			<0.001	N/A	6.6
Low Risk	56,286 (48)	103 (32)			
Moderate Risk	51,306 (44)	162 (51)			
High Risk	9,261 (8)	53 (17)			
Smoking	16,302 (13)	56 (16)	0.108	1.3 (1.0–1.7)	0
Alcohol intake	8,898 (7)	30 (9)	0.298	1.2 (0.8–1.8)	0
Myocardial infarction	89 (0)	1 (0)	0.223	4.0 (0.6–30.0)	0
COPD	6,979 (6)	52 (15)	<0.001	3.0 (2.2–4.0)	0
Liver disease	7,626 (6)	37 (11)	0.001	1.8 (1.3–2.6)	0
Renal disease	10,670 (9)	70 (20)	<0.001	2.7 (2.1–3.5)	0
Congestive heart failure	3,664 (3)	26 (7)	<0.001	2.7 (1.8–4.0)	0
Neurological disease	16,861 (13)	68 (19)	0.002	1.5 (1.2–2.0)	0
Seizures	3,863 (3)	15 (4)	0.151	1.5 (0.9–2.5)	0
Cancer	34,951 (28)	112 (32)	0.094	1.2 (1.0–1.5)	0
Steroid treatment	5,049 (4)	36 (10)	<0.001	2.7 (1.9–3.9)	0
Pneumonia	4,150 (3)	31 (9)	<0.001	2.8 (2.0–4.1)	0
Pulmonary hypertension	1,654 (1)	13 (4)	0.001	2.9 (1.7–5.0)	0
Coma	914 (1)	5 (1)	0.117	2.0 (0.8–4.8)	0
Sepsis	959 (1)	9 (3)	0.002	3.4 (1.8–6.6)	0
ASA physical status			<0.001	4.1 (2.9–5.7)	0.7
Class 1, 2 or 3	120,717 (97)	305 (89)			
Class 4 or 5	3,799 (3)	39 (11)			
Pre-operative Opioid	31,604 (25)	103 (29)	0.083	1.2 (1.0–1.6)	0
Pre-operative Benzodiazepine	14,085 (11)	51 (15)	0.059	1.3 (1.0–1.8)	0
***Admission Status***			<0.001	N/A	
Outpatient	64,047 (51)	87 (25)			
Admit on day of surgery	45,458 (36)	160 (46)			
Preoperative admission	15,884 (13)	104 (30)			
***Procedural risk factors***					
Anesthesia duration ≥ 75^th^ centile	36,562 (29)	190 (54)	<0.001	2.9 (2.3–3.5)	0
Intraoperative aminoglycoside	12,953 (10)	45 (13)	0.132	1.3 (0.9–1.7)	0
Duration of MAC above 1 ≥ 75^th^ centile	58,563 (47)	91 (26)	<0.001	0.4 (0.3–0.5)	0
***NMBA administered***			<0.001	N/A	
No NMBA administered	49,612 (39)	62 (18)			
NMB with reversal agent administration	74,899 (60)	285 (81)			
NMB without reversal agent administration	878 (1)	4 (1)			
Cisatracurium	7,331 (6)	25 (7)			0
Rocuronium	5,616 (5)	35 (10)			0
Vecuronium	62,830 (50)	229 (65)			0
NMBA dose ≥75^th^ centile	19,047 (15)	47 (13)	0.371	0.9 (0.6–1.2)	0
High dose Cisatracurium	1,805 (1)	5 (1)	0.999	1.0 (0.4–2.4)	0
High dose Vecuronium	16,381 (13)	43 (12)	0.686	0.9 (0.7–1.3)	0
High dose Rocuronium	1,278 (1)	13 (4)	<0.001	3.7 (2.1–6.5)	0
Any TOF use	46,318 (37)	164 (47)	<0.001	1.5 (1.2–1.8)	0
PRBC administration	1,944 (2)	17 (5)	<0.001	3.2 (2.0–5.3)	0
FFP administration	496 (0)	3 (1)	0.164	2.2 (0.7–6.8)	0
***Intraoperative opioid***					
Fentanyl	116,880 (93)	310 (88)	0.001	0.6 (0.4–0.8)	0
Hydromorphone	5,655 (5)	17 (5)	0.714	1.1 (0.7–1.8)	0
Morphine	33,393 (27)	100 (29)	0.433	1.1 (0.9–1.4)	0
***Anesthesia technique***			0.275	1.2 (0.8–1.7)	0
General Anesthesia	114,911 (92)	316 (90)			
General + Regional Anesthesia	10,478 (8)	35 (10)			
***Anesthesia Group Used***			<0.001	N/A	0.6
Propofol Infusion	16,415 (13)	27 (8)			
Volatile Anesthetic	96,861 (77)	205 (58)			
Combined propofol infusion and volatile anesthetic	11,335 (9)	119 (34)			
***Number of intravenous opioids***			0.006	N/A	0
0	5,278 (4)	25 (7)			
1	78,281 (62)	189 (54)			
2	40,518 (32)	133 (38)			
3	1,302 (1)	4 (1)			
4	10 (0)	0 (0)			
Intraoperative morphine equivalents ≥ 75^th^ centile	13,659 (11)	16 (5)	<0.001	0.4 (0.2–0.6)	0
Naloxone	747 (1)	19 (5)	<0.001	9.5 (6.0–15.2)	0
PCA	170 (0)	5 (1)	<0.001	10.6 (4.3–26.1)	0
High risk surgery	48,238 (39)	197 (56)	<0.001	2.0 (1.7–2.5)	0.5

Univariate unadjusted analysis revealed significant differences in the distribution of study variables across the outcome and control groups (Table 1). The following conditions were more frequent among patients who developed PRC: higher PSAP score categories, chronic obstructive pulmonary disease, liver disease, renal disease, congestive heart failure, neurological conditions, steroid use, pneumonia, pulmonary hypertension, sepsis, higher ASA physical status and admission status. There were significant differences in most of the intraoperative anesthesia interventions, with the exception of high dose NMBA, number of fresh frozen plasma units, morphine, and hydromorphone usage.

### Impact of desaturation measures

Median PACU SpO_2_ [AOR 1.1; 95% CI 0.77, 1.51; p = 0.650] and duration of desaturation below median [AOR 1.10; 95% CI 0.8, 1.52; p = 0.542] were not associated with the study outcome. Nadir desaturation below 10^th^ centile [AOR 2.02; 95% CI 1.52, 2.68; p<0.001] was identified as a PACU desaturation measure independently associated with early PRC, and was considered the primary exposure variable for the study. In addition, duration of oxygen therapy in PACU was also independently associated with early PRC with increasing adjusted odds across increasing quartiles of oxygen treatment duration [AOR range from 1.92 to 3.04; p<0.001]. The duration quartiles of oxygen therapy in PACU were also introduced as an ordinal variable in the primary models.

### Interaction between surgical service and early postoperative desaturation

Surgical procedure classified as high risk procedures based on the included CPT codes for surgical interventions involving intracranial, intrathoracic, spinal cord, upper abdominal, or lower abdominal body locations, radiological procedures and surgery for burns. Low risk surgery CPT codes involved CPT codes for surgical interventions involving neck, extrathoracic, perineal, pelvic, upper leg or knee, popliteal, lower leg, shoulder, upper arm, forearm and other body locations. Suspected OSA interacted significantly with higher surgical risk as shown in Table 2, and accordingly, stratified analyses were performed.

**Table 2:** Interaction between PSAP score and surgical risk.

Nadir desaturation	Lower risk surgery		Higher risk surgery	
	AOR	p-value	AOR	p-value
<89%	0.9 [0.6, 1.2]	0.467	1.7 [1.2, 2.4]	0.006
≥89%	0.3 [0.2, 0.4]	<0.001	0.5 [0.4, 0.8]	<0.001

Estimates are from a logistic regression model exploring the interaction between nadir desaturation and surgical risk.

#### Higher surgical risk model

Nadir desaturation [AOR 2.0; 95% CI 1.4, 3.0; p<0.001] and highest quartile of duration of oxygen therapy in PACU quartile [AOR 3.2; 95% CI 1.3, 7.9; p = 0.012] were independently associated with early PRC. Other risk factors associated with the outcome included PSAP score, renal disease, steroid use, anesthesia duration, duration of anesthesia exposure >1 MAC, reversal use, number of opioids, patient controlled analgesia, naloxone administration, and general anesthetic agent used (Table 3).

**Table 3:** Independent predictors of early postoperative tracheal intubation–Higher surgical risk.

	AOR	95% C.I.		p-value
		Lower	Upper	
Nadir Desaturation <10^th^ centile	2.0	1.4	3.0	<0.001
***PACU oxygen treatment duration quartiles***				
<30 minutes				
31–60 minutes	1.9	0.7	4.8	0.180
61–90 minutes	2.5	1.0	6.3	0.048
>90 minutes	3.2	1.3	7.9	0.012
***PSAP Score (OSA risk)***				
Low risk (PSAP 1–3) Baseline				
Moderate risk (PSAP 4–5)	2.3	1.4	3.6	<0.001
High risk (PSAP >5)	2.3	1.2	4.3	0.009
**Preoperative conditions**				
COPD	2.5	1.6	3.9	<0.001
Renal disease	1.7	1.1	2.7	0.019
Steroid treatment	1.9	1.2	2.9	0.049
**Procedural conditions**				
***Admission status***				
Outpatient Baseline				
Admit on day of surgery	0.8	0.5	1.4	0.510
Preoperative admission	1.4	0.8	2.6	0.236
Anesthesia duration ≥ 75^th^ centile	1.9	1.2	2.9	0.005
≥ 75^th^ centile of duration of MAC above 1	0.4	0.3	0.7	<0.001
Reversal use	2.6	1.4	4.8	0.003
Number of intravenous opioids	0.7	0.5	0.9	0.019
Patient controlled analgesia	10.5	3.0	36.7	<0.001
Naloxone administration	8.5	4.0	18.0	<0.001
***Anesthesia Group Used***				
Propofol Infusion (Baseline)				
Volatile Anesthetic	0.2	0.1	0.4	<0.001
Combined propofol infusion and volatile anesthetic	1.0	0.5	2.1	0.897
Anesthesia Technique General + Regional	0.5	0.3	1.0	0.059

Variables dropped by the model included neurological disease, pneumonia, pulmonary hypertension, sepsis, ASA status, use or non-use of NMBA, morphine equivalent dose, TOF use, high dose rocuronium, admit type and number of PRBCs.

#### Lower surgical risk model

Nadir desaturation [AOR 2.1; 95% CI 1.4, 3.1; p = 0.001] and the two highest oxygen treatment duration quartiles [AOR 3.0; 95% CI 1.4, 6.2; p = 0.003] were independently associated with early PRC. Other risk factors associated with the outcome included renal disease, admission status, anesthesia duration, duration of anesthesia exposure >1 MAC, high dose rocuronium and general anesthetic agent used (Table 4).

**Table 4:** Independent predictors of early postoperative tracheal intubation—Lower surgical risk.

	AOR	95% C.I.		p-value
		Lower	Upper	
Nadir Desaturation 10^th^ centile	2.1	1.4	3.1	0.001
***PACU oxygen treatment duration quartiles***				
<30 minutes				
31–60 minutes	2.0	0.9	4.2	0.069
61–90 minutes	3.0	1.5	6.2	0.003
>90 minutes	3.0	1.4	6.2	0.003
**Preoperative conditions**				
Renal disease	2.6	1.6	4.2	<0.001
**Procedural conditions**				
***Admission status***				
Outpatient Baseline				
Admit on day of surgery	1.1	0.7	1.8	0.661
Preoperative admission	1.9	1.1	3.3	0.028
Anesthesia duration ≥ 75^th^ centile	2.6	1.7	4.0	<0.001
≥ 75^th^ centile of duration of MAC above 1	0.6	0.4	0.9	0.006
***Anesthesia Group Used***				
Propofol Infusion (Baseline)				
Volatile Anesthetic	0.8	0.4	1.7	0.598
Combined propofol infusion and volatile anesthetic	2.8	1.3	5.9	0.008
High dose Rocuronium	5.2	2.1	12.4	<0.001

Variables dropped by the model included PSAP risk, COPD, liver disease, congestive heart failure, neurological disease, steroid treatment, pneumonia, pulmonary hypertension, sepsis, ASA status, reversal use, PCA use, naloxone use, number of opioids, morphine equivalent dose, TOF use,number of PRBCs, and NMB administered.

#### Model diagnostics

The c-statistics [±SE] of the stratified models were 0.79 [±0.02] and 0.77 [±0.02]. The variance inflation factors were below 10 and condition indices of test variables were all below 30. The omnibus test of model coefficients revealed a chi-square of 134.832 for the lower surgical risk model with 12 degrees of freedom and 231.984 for the higher surgical risk model with 20 degrees of freedom and p <0.001.

#### Secondary outcomes

A good match balance was unable to be achieved with the nadir desaturation variable. In order to identify which quartile of duration of oxygen therapy in PACU should serve as the threshold for matching cohorts, we examined the raw relationship between oxygen therapy duration quartiles and overall charges, The biggest step increase in median charges was noted between the first and next higher quartiles ($10873, $12054, $12678, and $13203 across lowest to highest quartiles of duration of oxygen therapy in PACU). Therefore, we successfully attempted to match balance on the lowest quartile of oxygen treatment duration, since this variable is also independently associated with the study outcome. Table 5 describes the distribution of pre-match and post-match cohorts.

**Table 5:** Unmatched and matched univariates.

	Unmatched (N = 107,196)			Matched (N = 37,354)		
	*Oxygen treatment duration>30 minutes (N = 27*,*632)*	*Oxygen treatment duration≤30 minutes (N = 79*,*564)*	*p-value*	*Oxygen treatment duration>30 minutes (N = 18*,*677)*	*Oxygen treatment duration≤30 minutes (N = 18*,*677)*	*p-value*
Risk for OSA (PSAP categories)			<0.001			0.276
Low Risk	31,311 (42)	17,459 (66)		11,149 (60)	11,014 (59)	
Moderate Risk	35,814 (48)	8,044 (31)		6,776 (36)	6,925 (37)	
High Risk	7,124 (10)	762 (3)		752 (4)	738 (4)	
***Preoperative Conditions***						
COPD	5,078 (6)	651 (2)	<0.001	517 (3)	548 (3)	0.335
Liver Disease	4,804 (6)	957 (3)	<0.001	800 (4)	786 (4)	0.719
Renal Disease	7,701 (10)	1,099 (4)	<0.001	974 (5)	961 (5)	0.762
Congestive Heart Failure	2,653 (3)	255 (1)	<0.001	248 (1)	233 (1)	0.491
Neurological Disease	11,712 (15)	2,261 (8)	<0.001	1,792 (10)	1,754 (9)	0.502
Steroid Treatment	3,484 (4)	621 (2)	<0.001	583 (3)	532 (3)	0.121
Pneumonia	2,775 (3)	482 (2)	<0.001	398 (2)	387 (2)	0.692
Pulmonary Hypertension	1,195 (2)	127 (0)	<0.001	132 (1)	117 (1)	0.340
Sepsis	620 (1)	101 (0)	<0.001	90 (0)	87 (0)	0.821
ASA physical status			<0.001			0.684
Class 1, 2 or 3	76,448 (97)	27,277 (99)		18,478 (99)	18,486 (99)	
Class 4 or 5	2,580 (3)	218 (1)		199 (1)	191 (1)	
***Admission Status***			<0.001			0.020
Outpatient	31,195 (39)	22,854 (83)		14,079 (75)	14,302 (77)	
Admit on day of surgery	37,484 (47)	3,526 (13)		3,466 (19)	3,270 (18)	
Preoperative admission	10,885 (14)	1,252 (5)		1,132 (6)	1,105 (6)	
***Anesthesia duration quartiles***						
≥ 75^th^ centile	28,233 (35)	3,594 (13)	<0.001	3,093 (17)	3,023 (16)	0.328
***Anesthesia Group Used***			<0.001			0.025
Propofol Infusion	5,878 (7)	9,387 (34)		3,918 (21)	3,707 (20)	
Volatile Anesthetic	66,213 (84)	15,559 (57)		12,877 (69)	13,046 (70)	
Combined propofol infusion and volatile anesthetic	7,123 (9)	2,498 (9)		1,882 (10)	1,924 (10)	
***Quartile of duration of MAC above 1***						
≥ 75^th^ centile	36,822 (46)	13,960 (51)	<0.001	9,060 (49)	9,111 (49)	0.600
***NMBA administered***			<0.001			0.306
No NMBA used	24,585 (31)	16,053 (58)		9,641 (52)	9,669 (52)	
Cisatracurium	5,298 (7)	584 (2)		575 (3)	524 (3)	
Rocuronium	2,740 (3)	1,660 (6)		917 (5)	964 (5)	
Vecuronium	46,941 (59)	9,335 (34)		7,544 (40)	7,520 (40)	
Reversal use	53,104 (67)	10,763 (39)	<0.001	8,532 (46)	8,487 (45)	0.640
PRBC Administration	1,282 (2)	83 (0)	<0.001	80 (0)	75 (0)	0.687
Fentanyl	74,785 (94)	25,321 (92)	<0.001	17,301 (93)	17,319 (93)	0.721
Naloxone	603 (1)	69 (0)	<0.001	54 (0)	52 (0)	0.846
***Number of intravenous opioids***			<0.001			<0.001
0	2,646 (3)	1,653 (6)		966 (5)	879 (5)	
1	43,866 (55)	21,234 (77)		13,680 (73)	13,933 (75)	
2	32,065 (40)	4,562 (17)		3,940 (21)	3,715 (20)	
3	981 (1)	181 (1)		90 (0)	149 (1)	
4	6 (0)	2 (0)		1 (0)	1 (0)	
***Quartiles of intraoperative morphine equivalents***						
≥ 75^th^ centile	8,167 (10)	3,637 (13)	<0.001	2,484 (13)	2,503 (13)	0.773
PCA	115 (0)	7 (0)	<0.001	10 (0)	6 (0)	0.317
TOF use	7,180 (9)	1,291 (5)	<0.001	1,143 (6)	1,125 (6)	0.697
High Dose Rocuronium	717 (1)	306 (1)	0.002	187 (1)	207 (1)	0.311
***Surgery type by CPT code***						
Head	8,661 (11)	3,259 (12)	<0.001	2,396 (13)	2,497 (13)	0.121
Neck	8,986 (11)	1,756 (6)	<0.001	1,517 (8)	1,494 (8)	0.662
Thorax (Chest wall and shoulder girdle)	5,226 (7)	3,256 (12)	<0.001	1,968 (11)	1,949 (10)	0.748
Intrathoracic	2,632 (3)	153 (1)	<0.001	161 (1)	141 (1)	0.248
Spine and spinal cord	3,249 (4)	301 (1)	<0.001	288 (2)	280 (2)	0.735
Upper abdomen	9,033 (11)	1,272 (5)	<0.001	1,063 (6)	1,086 (6)	0.609
Lower abdomen	12,358 (16)	3,112 (11)	<0.001	2,304 (12)	2,293 (12)	0.863
Perineum	9,284 (12)	4,701 (17)	<0.001	3,346 (18)	3,348 (18)	0.979
Pelvis (except hip)	411 (1)	92 (0)	<0.001	76 (0)	78 (0)	0.872
Upper leg (except knee)	3,497 (4)	609 (2)	<0.001	453 (2)	461 (2)	0.789
Knee and popliteal area	2,778 (3)	2,611 (9)	<0.001	987 (5)	941 (5)	0.282
Lower leg (below knee, including ankle and foot)	2,937 (4)	1,430 (5)	<0.001	918 (5)	898 (5)	0.630
Shoulder and axilla	2,754 (3)	2,607 (9)	<0.001	1,377 (7)	1,371 (7)	0.905
Upper arm and elbow	638 (1)	303 (1)	<0.001	220 (1)	209 (1)	0.593
Forearm, wrist and hand	1,477 (2)	816 (3)	<0.001	519 (3)	546 (3)	0.401
Radiological procedure	3,108 (4)	540 (2)	<0.001	465 (2)	460 (2)	0.868
Burn excisions or debridement	228 (0)	46 (0)	<0.001	44 (0)	39 (0)	0.583
Obstetric	108 (0)	117 (0)	<0.001	80 (0)	92 (0)	0.359
Other procedure	57 (0)	10 (0)	0.042	13 (0)	5 (0)	0.059
Missing	2,142 (3)	641 (2)	<0.001	482 (3)	489 (3)	0.820

Post-match diagnostics revealed that resource utilization as measured by length of stay, day of surgery charges, surgery charges, respiratory charges and total charges were significantly higher in the lowest quartile of oxygen treatment duration group. Patients in the matched exposure cohort had higher odds of developing early PRC [odds ratio 2.5; 95% CI 1.3, 5.0; p = 0.007], and needing invasive or non-invasive ventilatory support [odds ratio 5.0; 95% CI 3.3, 5.0; p<0.001] (Table 6). Table 7 describes total charges across the four PACU oxygen therapy quartiles in both unmatched and matched cohorts.

**Table 6:** Secondary outcomes on matched dataset.

	*Oxygen treatment duration>30 minutes* (N = 18,677)	Oxygen treatment duration≤30 minutes (N = 18,677)	p-value
**Total Charges**			
Charge (median, 25^th^, 75^th^ centile)	12,532 [8,683 to 21,690]	10,874 [7,661 to 18,190]	<0.001
**Day of Surgery Charges**			
Charge (median, 25^th^, 75^th^ centile)	9,749 [5,001 to 15,076]	8,672 [4,623 to 13,370]	<0.001
**Surgery Charges**			
Charge (median, 25^th^, 75^th^ centile)	6,075 [0 to 10,508]	6,205 [0 to 10,162]	<0.001
**Respiratory Charges**			
Charge (median, 25^th^, 75^th^ centile)	0 [0 to 370]	0 [0 to 125]	<0.001
**Hospital Length of Stay** (median, 25^th^, 75^th^ centile)	0 [0 to 1]	0 [0 to 1]	<0.001
**Early PRC**			
Reintubation [n(%)]	34 (0)	15 (0)	0.007
Ventilatory support [n(%)]	551 (3)	131 (1)	<0.001

All data are reported as either frequency (percent) or median [25^th^ percentile to 75^th^ percentile], as appropriate.

**Table 7:** Total charge data across PACU oxygen therapy quartiles.

PACU oxygen therapy quartiles	Unmatched cohort—total charges in dollars				
	Median	Percentile 25	Percentile 75	Valid N	Total N
<30 minutes	10187.00	7360.05	15652.60	20671	27632
31–60 minutes	20354.00	11375.55	40504.46	22037	26254
61–90 minutes	23955.77	12646.31	42348.34	23128	26370
>90 minutes	23814.44	13454.05	39598.41	24344	26940
	Matched cohort—total charges in dollars				
	Median	Percentile 25	Percentile 75	Valid N	Total N
<30 minutes	10873.85	7660.74	18189.16	14499	18677
31–60 minutes	12054.95	8639.41	18706.39	5594	7472
61–90 minutes	12678.46	8767.35	23374.65	4715	6053
>90 minutes	13203.71	8613.03	24989.58	4164	5152

## Discussion

Early postoperative desaturation as measured by the nadir desaturation <89% or longer duration of oxygen requirement in PACU is independently associated with increased risk of early PRC and resource utilization. Surgical procedure risk modestly influences this relationship.

Expert guidelines suggest using early postoperative desaturation as a trigger for identifying patients at higher risk of postoperative respiratory complications, but there are limited data to support this until this study.[27] The early postoperative period captures the most intense clinical interaction between patients and healthcare providers, especially in PACU, where nursing ratios are at least 1:2, and often 1:1. Traditional desaturation measures in sleep medicine are based on short (10 seconds or more) episodes of relative changes in SpO_2_. This has very little resemblance to the way SpO_2_ monitoring is used postoperatively. Pulse oximetry devices typically average the signal once every 6–8 seconds, and threshold alarms are set anywhere between 80–90% depending on the unit or institution. In contrast, the research database captures a sampled SpO_2_ value every minute. It is possible that nadir desaturation values for individual patients are transiently lower at times when the data are not recorded in the database. Despite this apparent shortcoming, we were able to demonstrate a clear data distinction between duration of desaturation below median PACU SpO_2_ levels and nadir desaturation. Our intent with these measures was to separate patients into distinct risk groups based on each measure, since we believe that each desaturation measure represents a certain phenotype of patient. Patients who have nadir desaturation data below the 10% centile for population spread of nadir desaturation values in PACU may be considered as those who are presenting more severe episodic desaturation events, when compared with other patients above the 10^th^ centile of nadir desaturation in this study. The population threshold value for nadir desaturation was 89% which incidentally is considered by sleep medicine experts and the CMS as the threshold saturation that defines the need for oxygen therapy. Thus, we consider that this nadir saturation value has reasonable clinical relevance.

This study also highlights the importance of measuring the duration of oxygen therapy as an independent risk factor for developing PRC. Indeed patients who require oxygen therapy for ≥75% of PACU time (or greater 90 min) than appear to be at greater risk of developing early PRC than nadir desaturation. This fact may be explained by the fact that oxygen therapy tends to ameliorate but not abolish the occurrence of desaturation.[14,28] This may also reflect a masking of ongoing hypoventilation during oxygen therapy.[29] It is therefore interesting that we have identified longer oxygen therapy requirements in PACU as an independent risk factor for step-wise increaser in odds of early PRC. The reduction of desaturations that one expects to see with oxygen therapy does not seem to alter the underlying risk state, further supporting our research strategy to classify patients based on duration of oxygen therapy in PACU.

Up to 50% of postoperative patients demonstrate episodic hypoxemia in the absence of oxygen therapy.[14] In our study, 14% of patients had a threshold nadir desaturation event, while 26% of patients had oxygen requirements for over 75% of their PACU time. One of the major causes for postoperative desaturation is OSA, with over 24% of middle aged males 9% of middle aged women having the condition.[30] This study was focused on the immediate postoperative period, since this is when we collect the most frequent oximetry data in our database. The frequency of data sampling in our study is at every 1 min, making any comparison with polysomnographic data impossible, since the data are sampled at least every second for defining AHI. OSA is associated with increase in the number and severity of desaturation episodes—this is the diagnostic definition of the disease condition. These episodes may last for any duration over 10 seconds, but the majority of such events are self-terminated before a minute. This means that postoperative desaturation may occur in patients with OSA, but sampling at 1 minute (as we and other researchers in this area have done) may miss the majority of such events. The adverse influence of OSA on morbidity has been investigated for the last decade.[8,31–36] Increasing severity of sleep disordered breathing is associated with increased risk of complications after surgery.[37] This may indeed explain the association of increased early PRC seen with longer PACU oxygen requirement in the propensity matched analyses. This outcome association occurred despite the fact that suspected OSA as screened by the PSAP score was well balanced across the matched groups. This may suggest that postoperative desaturation is an additional screening test for patients with OSA that are missed by the screening test. Indeed, preoperative episodic hypoxemia is predictive of both presence of OSA[38] and the occurrence of postoperative hypoxemia.[39] Alternatively postoperative desaturation may indicate the development of postoperative pulmonary complications that required additional respiratory therapy through invasive or non-invasive ventilation as we observed in our study.

In this study, several anesthesia variables were evaluated as independent risk factors. The data reflect anesthetic choices made by clinicians in a large diverse department. The use of certain drugs may reflect knowledge of greater risk of adverse event and so may be at risk for indication bias. It was beyond our scope of a single manuscript to evaluate the interaction between each of these interventions and the occurrence of PACU desaturation or PRC. We have introduced these interventions into the logistic regression models as a way to adjust for the relationship between PACU desaturation and PRC. Anesthesia technique, duration of exposure to anesthesia, patient controlled analgesia, naloxone usage, neostigmine reversal and use of high dose rocuronium were all associated with the occurrence of early PRC. Longer anesthesia times but not duration of exposure of general anesthesia MAC levels >1 were associated with early PRC. This finding is likely reflective of longer surgical procedures conferring the risk, rather than the anesthetic agents themselves. Indeed the strongly protective relationship between anesthetic agent exposure and early PRC suggests that we are measuring a clinical bias towards lower general anesthesia MAC in sicker patients at greater risk of the study outcome. The surprising increase in odds of early PRC with reversal use is most likely a reflection of use of NMBAs themselves, since >97% of patients who received NMBAs also received reversal. There may be alternative explanations for this occurrence through inappropriate reversal management.[40]

The increased resource utilization in patients with longer oxygen therapy requirement in PACU likely reflects the increase in occurrence of PRC requiring invasive and non-invasive ventilatory support, especially on the day of surgery, since this appears to account for the most significant difference between the matched groups. It is also important to note that the increased charges were not driven only by outliers, but were observed to uniformly rise along with increasing durations of oxygen therapy. It is now well established that oxygen therapy in the postoperative period significantly reduces abnormal desaturation episodes seen with sleep disordered breathing.[41] This finding may partially explain the lack of relationship between desaturation measures and STOP-Bang scores or opioid type in other studies.[42,43] The association of oxygen therapy with increased resource utilization and PRC suggests that such treatment improves oxygenation but does not alter the underlying risk of the patient for adverse outcome.

One of the significant challenges in this study was how best to handle oximetry data. To the best of our knowledge, there is no established standard of describing postoperative oximetry data sampled at 1 minute. Indeed our study is the first to describe a clinically relevant relationship between two of the measures we describe and PRC/resource utilization. Khanna et al have shown that the use of integrated area under the curve of saturation of 90% per h using median quantile regression is one way of describing both dimensions of severity and duration of desaturation, when all postoperative data are taken as a whole.[42,43] We did describe this concept in a simpler fashion by presenting both the central tendency measure (median of medians) and duration of desaturation below the median threshold of 94%. Tarassenko and colleagues have previously shown in multiple studies that it is feasible to convert continuous physiological data (5 vital signs including oximetry) into threshold variables for the purposes of creating clinically meaningful early warning systems.[44,45] Similar to Tarassenko, we chose to convert continuous SpO_2_ data into categorical variables by defining thresholds of population spread to describe a generalizable method that may be adapted into clinical practice without need for advanced statistical processes. Based on the population distribution of PACU oximetry data from patients included in this study, we described individual measures of central tendency and severity of desaturation. Tarassenko’s group proposed the use of 3SD (0.3rd and 99.7th centile) as the population threshold, which is reasonable when analyzing 96 hours of data to determine abnormal values. We had a maximum of 120 minutes (data points) of SpO_2_ data per patient to develop our measures, and so chose less stringent thresholds of 10th centile (for capturing lower limit of desaturation measures) or 90th centile (for capturing upper limit of duration). Interestingly, they identified a median SpO2 of 94% from their data, similar to our study.

There exists the possibility of bias from misclassification of both our primary health outcome and variables of interest in our study. In order to minimize misclassification bias, objective measures were used to define the outcome and covariates whenever possible. Specifically, for SpO_2_ data, we employed a 60% lower limit for two reasons: pulse oximetry is not validated below this cut-off value and sensor dislodgement artifacts are more likely to occur when lower values are displayed. We defined respiratory failure as the need for reintubation in the early postoperative period. Since we have a variable process of determining eligibility for non-invasive ventilation (OSA patient bringing their home device for usual application vs. patient in respiratory failure trialing noninvasive ventilation), we chose a more conservative end-point of significant PRC needing tracheal intubation in order to ensure that the outcome is standardized across all included patients. Retrospective studies may also be prone to selection bias, because the outcome has already occurred and risk factors are known before study inclusion. This could lead to selective grouping to maximize study findings. We have used the PSAP score as a way to adjust for OSA-risk phenotype. This approach has inherent selection bias since there is an implicit acceptance of the diagnostic error with screening tools such as PSAP or STOP-Bang. The alternate approach of using only formally diagnosed OSA patients, would wrongly miss at least 50% of patients with the condition, introducing a different selection bias into the study. The propensity matched analyses was performed to limit confounding from many such factors. In this case, the observational dataset has many inherent limitations. Because of the nature of retrospective studies, there exists a lack of randomization and subsequent biases are introduced into the observational design. Furthermore, in this particular study, there may be issues related to misclassification of both the exposure variable and confounders. The study may not be generalizable to other hospitals or postoperative patient care locations. The PACU reflects an environment where nurse-patient ratios are amongst the highest in the hospital. It is also the place where nurses frequently intervene with respect to opioid analgesia therapy. It is our assumption that this environment imparts a bias to the data, because such nursing presence would limit desaturation episodes. The influence of inspired oxygen concentration on outcomes could not be evaluated in this study, since such data are not part of the research or clinical database. Our approach to the occurrence of ongoing requirement for oxygen was to consider it reflective of a certain risk state: either one where the patient is demonstrating ongoing desaturation or one where the nurse is concerned about potential for future desaturation during PACU or post-PACU care. Finally, the binary categorization of surgical risk is unusual. Our intent was to ensure that we evaluated the desaturation measures across different surgical risk, without making the study overtly complicated.

Despite these limitations, our study provides a framework for using retrospective SpO_2_ data in an AIMS database to enrich risk assessment methods. We also identified the occurrence of nadir desaturation <89% and longer oxygen requirements in PACU as potential phenotypic markers that may be modifiable. There are resource implications with the occurrence of PACU desaturation, justifying the need for further research to differentiate whether this is an intermediate outcome in the continuum of early PRC and if the outcome can be modified by reducing PACU desaturation risk.

## IRB

This study was approved by the University of Michigan's Institutional Review Board (project number HUM00069035) [Contact: 734-763-4768 or at irbmed@umich.edu]

## Supporting information

S1 Appendix Study variables. (DOCX)
