# Supplementary material for: Retrospective observational evaluation of postoperative oxygen saturation levels and associated postoperative respiratory complications and hospital resource utilization
Source: PLoS One. 2017 May 17;12(5):e0175408. doi: 10.1371/journal.pone.0175408 (PMC5435138; doi:10.1371/journal.pone.0175408)
Supplement: S1 Appendix — (DOCX) [file pone.0175408.s001.docx]

## S1 Appendix – study variables

**Patient Characteristics and Risk Factors**

**POSTOPERATIVE DESATURATION**

-Dataset Location= local UMHS MPOG database

-Variable Name= 50452 Postoperative vital signs – SpO2

-Variable Type= Continuous

Postoperative SpO2 is captured every minute during the PACU stay within the PACU database. There are several validated measures of desaturation in sleep literature, but they are described for higher frequency data. Literature reviews on desaturation report an extremely large spectrum of definitions as listed. This lack of homogeneity in definition has major clinical and legal implications. On a practical level, this definition does not stipulate the duration or frequency of the inadequate response.[[22](#_ENREF_22)] Additionally, the uniqueness of SpO2 data in highly monitored environments is that they tend to be addressed either by patient arousals secondary to alarm noise, or by nurse intervention. These interventions range from stimulating the patient to increasing the inspired oxygen fraction. So, it is anticipated that the majority of SpO2 values (>95%) will fall in the normal range, and examining the 10^th^ or 90^th^ centile thresholds may be necessary to better quantify variance across study data. Additionally, it is also esential to examine desaturation across dimensions of duration, central tendency and nadir levels of desaturation. Accordingly, the following measures of desaturation will be evaluated in multiple sensitivity analyses to validate the best measure for outcome prediction.

CENTRAL TENDENCY MEASURES

- Median of SpO2 in PACU
  - Median SpO2 value during PACU stay
    - Starting from first recording of SpO2 in the PACU “Vitals” tab, through to PACU discharge, all SpO2 values in the entire period will be used for calculating the median SpO2
  - Median values for a q 1min sliding window of 5 minute durations will be used to determine the median values. On a per patient basis, the median of these derived values will be determined across all sliding window medians. All median data will be transformed into a dichotomous measure of central tendency of desaturation by determining the population spread of these data, and identifying the 10^th^ centile of desaturation medians, since lower median values likely signify greater respiratory risk. Values at and below the 10^th^ centile of median values vs. those above the 10^th^ centile will be considered as a dichotomous variable.

DURATION OF DESATURATION:

- The cumulative time in minutes with SpO2 below median value, will be expressed as number of minutes per hour of PACU SpO2 monitoring. All duration data will be transformed into a dichotomous measure of duration of desaturation by determining the population spread of these data, and identifying the 90^th^ centile of desaturation duration, since longer desaturation periods likely signify greater respiratory risk.

NADIR DESATURATION MEASURES”

- Minimum SpO2 on room air exposure will be chosen as the lowest value among the two following measures:
  - 5 min Room Air (%) in PACU “Resp” tab - SpO2 during 5 min room air exposure OR
  - Lowest SpO2 value during all continuous monitoring periods: Starting from first recording of SpO2 in the PACU “Vitals” tab, through to PACU discharge, all SpO2 values in the entire period will be used for calculating the median SpO2
- All nadir desaturation data will be transformed into a dichotomous measure of desaturation by determining the population spread of these data, and identifying the 10^th^ centile of desaturation nadirs, since lower nadir values likely signify greatest respiratory risk. Values at and below the 10^th^ centile of median values vs. those above the 10^th^ centile will be considered as a dichotomous variable.

DURATION OF ROOM AIR EXPOSURE IN PACU

-Dataset Location= local UMHS MPOG database

-Variable Name= PACU O2 settings - in PACU “Resp” tab

-Variable Type= Continuous

- These data are filled in on a 30-60 minute basis manually by PACU nurses, using a pick-list choice of “room air” or ­__ l/min of O2.
- The total duration of “room air” exposures will be extracted from these records.
- The exposure will be presented as a fraction of overall PACU time.

Each of the measures described above defines a specific characteristic of the desaturation, with some patients having shallow but prolonged desaturations and others with deep short epochs of deep desaturation. Similarly, the use of supplemental oxygen is associated with increased SpO2 levels and masking of hypoventilation. As we anticipate that the FiO2 will be titrated to effect by PACU RNs, we intend to use normalized duration of room air exposure to adjust for the effect of any desaturation measure in the outcome models.

**4. Variables and Epidemiological Measurements**

This study will be retrospective and observational in design. Two main sources will be used to collect patient data: The Centricity Anesthesia Information Management System and local UMHS MPOG data.

4.2 Outcomes –

**The primary health outcomes of interest** for this study are postoperative reintubation in the early postoperative period.

Reintubation is coded in multiple ways in our database. This is entered either as a pick-list choice clinical event note entry in the intraoperative record, the postoperative visit record or an urgent airway management note. Additionally reintubation may also be recorded as syntax in the comments table of the database. A key secondary check will be performed for this outcome variable by ensuring that the reintubation occurred after a primary extubation.

- 1. **Immediate postoperative reintubation or intubation after anesthesia end (OR and PACU)**
     1. Dataset – Centricity > CDR, IOP data and MPOG
     2. Variable name – Any of the following:
        1. QA Clinical events (object_sys=19061 and value like %reintub% in the comments table)
        2. MPOG ID 90215 CPOM measure Reintubation – Outcome Observations
        3. MPOG ID 90215 CPOM measure Reintubation Outcome Observations

OR either of the two intubation variables

1. Centricity Object_Sys 20442 __ mm __ ET tube taped at __ cm
2. MPOG ID 50122 Intubation Endotracheal Tube Size

TIME STAMPED AFTER one of the following

- 1. Centricity Documented Extubation (Object_Sys 3314 in IOP data)
  2. MPOG ID 50127 Intubation Extubated Awake or Deep
  3. Centricity Anesthesia end (Object _Sys 3279 in IOP data)
  4. MPOG ID 50009 AACD Anesthesia End Date/Time

OR presence of LMA AFTER EXTUBATION

-Dataset Location= QA Data

-Variable Name= LMA after extubation

-Variable Type= Dichotomous

LMA after extubation will be reported as a dichotomous variable: yes/no for LMA after extubation. This can be considered a rescue intervention that reflects significant airway or ventilatory failure resulting in need for placement.

1. **Postoperative intubation occurring within 24hours of surgery**
   1. Dataset – Procedure Notes
      - Text syntax search for value %intubation%

**The secondary outcome of this study** is resource utilization as measured by length of stay in PACU (ambulatory surgery patients) and hospital length of stay (hospitalized patients). Ambulatory center patients are more likely to be discharged immediately on attaining discharge criteria, unlike patients at the University Hospital, and therefore would be a better target population for examining the effect of desaturation on PACU discharge times. On the other hand, hospitalized patients at the University Hospital would be the target for examining hospital length of stay. The purpose of the analysis will be to estimate the incremental healthcare resource use and costs associated with post-operative oxygen desaturation in the PACU. Measures of healthcare resource use and cost will include PACU length of stay, hospital length of stay, post-operative respiratory therapy charges, post-operative ICU charges and total post-operative inpatient charges. Post-operative oxygen desaturation can occur due to a variety of factors, including complications of surgery, intra-operative opioid use, patient underlying physical condition and disease, and residual neuromuscular blockade. This analysis will describe healthcare resource use and costs of post-operative oxygen desaturation due to any underlying cause.

-Dataset Location= Centricity (PACU length of stay); RDW (Hospital length of stay); RDW (Hospital charges)

-Variable Name= Length of stay, hospital charges (split into major charge categories)

-Variable Type= continuous

Data on the following variables will be collected from the RDW for each patient:

- Total charges of inpatient care,
- Total charges of care on day of surgery,
- Respiratory therapy charges
- Ventilatory charges (if available)
- Intensive care unit charges (if available)
- Pre-surgical charges (used to verify adequacy of propensity score matched groups)
- Pure surgical charges (used to verify adequacy of propensity score matched groups)
- Hospital-length-of-stay,

DEMOGRAPHICS, ANTHROPOMETRICS, AND COMORBIDITIES VARIABLES

**OSA risk status:** The following variables are components of the PSAP score. Patients meeting the inclusion criteria will be retrospectively classified into groups at low, moderate and high risk of OSA using PSAP score. The score is calculated as follows:

- Low risk of OSA = <3 variables
- Suspected OSA
  - Moderate risk of OSA = 3-5 variables
  - High risk of OSA = greater than or equal to 6 variables

The variables in PSAP score are: age>43 years, male gender, body mass index >30 kg/m2, history of snoring, history of type 2diabetes mellitus, history of hypertension, documented thick neck (subjective estimation), estimated thyromental distance <6 cm, and modified Mallampati score 3 or 4. These variables are not weighted as per the validation analysis published previously.^19^ The presence of moderate or high risk of OSA (PSAP > or =3 variables) will initially be included as an independent variable in the prediction model. Variables will be identified based upon the pre-defined MPOG, AIMS or Centricity data fields.

AGE

-Dataset Location= local UMHS MPOG database

-Variable Name= AIMS_Patient_Age_Years

-Variable Type= Both dichotomous and continuous

Age will be included (either 43 years and over, or under 43 years old) as a dichotomous outcome for the PSAP score. It will be a primary variable used to determine eligibility for the study, and is also one the variables used to determine PSAP score. Thus, age will be evaluated as a risk factor for our primary outcome.

GENDER

-Dataset Location= local UMHS MPOG database

-Variable Name= AIMS_Sex

-Variable Type= Dichotomous

Male gender is one of the factors included in PSAP score, and therefore may be associated with our health outcome of interest. Male gender (yes/no) will be considered a dichotomous outcome for the PSAP score.

BODY MASS INDEX

-Dataset Location= local UMHS MPOG database

-Variable Name= AIMS_Body_Mass_Index

-Variable Type= Categorical

To improve clinical usability, body mass index will be classified using the World Health Organization groups: underweight (less than 18.50 kg/m2), normal weight (18.50–24.99 kg/m2), overweight (25.00–29.99 kg/m2), obese class I (30.00–34.99 kg/m2), obese class II (35.00–39.99 kg/m2), and obese class III (>=40.00 kg/m2). Obesity class I and greater will be considered a dichotomous outcome for the PSAP score.

SNORING

-Dataset Location= Centricity

-Variable Name= Pulmonary>Snoring

-Variable Type= Dichotomous

Snoring will be considered a dichotomous (yes/no) outcome for the PSAP score

DIABETES MELLITUS TYPE II

-Dataset Location= Centricity

-Variable Name= Centricity>Endocrine>Diabetes>Type 2

- Variable Type= Dichotomous

Type 2 Diabetes is one of the factors included in PSAP score, and therefore may be associated with our health outcome of interest. It will be considered a dichotomous (yes/no) outcome for the PSAP score

HYPERTENSION

-Dataset Location= local UMHS MPOG database

-Variable Name= 70031 Cardiovascular – Hypertension

-Variable Type= Dichotomous

Patients with a diagnosis of hypertension will be identified as having the condition. It will be considered a dichotomous (yes/no) outcome for the PSAP score. Medications being used to treat existing hypertension will be included in the “home medications” variable.

NECK CIRCUMFERENCE

-Dataset Location= local UMHS MPOG database

-Variable Name= Neck_Anatomy

-Variable Type= Dichotomous

Neck circumference is one of the factors included in PSAP score, and therefore may be associated with our health outcome of interest. Documented estimation of presence of thick neck on anesthesia H&P will be considered a dichotomous (yes/no) outcome for the PSAP score.

MODIFIED MALLAMPATI SCORE

-Dataset Location= local UMHS MPOG database

-Variable Name=

70001 Airway - Mallampati Score

70007 Airway - Mallampati_Neutral

-Variable Type= Dichotomous (either 3 or 4, or not 3 or 4)

Mallampati Score is one of the factors included in PSAP score, and therefore may be associated with our health outcome of interest. Mallampati scores 3 and 4 on anesthesia H&P will be considered a dichotomous (yes/no) outcome for the PSAP score.

THYROMENTAL DISTANCE

-Dataset Location= local UMHS MPOG database

-Variable Name= Hyoid_to_Mentum_Subjective

-Variable Type= Dichotomous

Thyromental distance is one of the factors included in PSAP score, and therefore may be associated with our health outcome of interest. Estimated thyromental distance less than 6 cm on preoperative anesthesia H&P will be considered a dichotomous (yes/no) outcome for the PSAP score.

Additional Variables to be Analyzed

IDEAL BODY WEIGHT

- Dataset Location= local UMHS MPOG database

- Variable Name= MPOG_Ideal_Body_Weight

- Variable Type= Continuous

Ideal Body weight will be used to quantify tertile of intraoperative NMB dosage

CURRENT SMOKER

-Dataset Location= local UMHS MPOG database

-Variable Name= 70128 History - Tobacco

-Variable Type= Dichotomous

Smoking will be treated as a dichotomous variable, and evaluated for potential association with respiratory failure postoperatively.

ALCOHOL USE

-Dataset Location= local UMHS MPOG database

-Variable Name= 70126 History - Social History - Alcohol

-Variable Type= Dichotomous

While alcohol use has not been linked explicitly to our health outcome of interest, it is an important patient characteristic that may be a risk factor. Therefore, we are including alcohol use in our analysis as a dichotomous variable; low or no use versus moderate or high use.

DYSPNEA

-Dataset Location= Centricity Cardiovascular>cardiac symptoms>dyspnea at rest OR dyspnea on exertion OR orthopnea OR paroxysmal nocturnal dyspnea

Pulmonary>Symptoms>SOB

-Variable Name= 70040 Cardiac

-Variable Type= Dichotomous

Dyspnea is indicative of respiratory health problems, and may therefore be linked to PRC. Patients with a history of dyspnea will be identified as having the condition. The disease will be treated as a dichotomous variable, with the patient being flagged as either having or not having the condition.

PULMONARY HYPERTENSION

-Dataset Location= Centricity>Pulmonary> Other – pulm>pulmonary HTN OR MPOG

-Variable Name= 70036 Cardiovascular - Pulmonary Hypertension

-Variable Type= Dichotomous

Pulmonary hypertension is indicative of respiratory health problems, and may therefore be linked to PRC. Patients with a diagnosis of pulmonary hypertension will be identified as having the condition. The disease will be treated as a dichotomous variable, with the patient being flagged as either having or not having the condition.

COPD

-Dataset Location= local UMHS MPOG database

-Variable Name= 70115 Respiratory - COPD General

-Variable Type= Dichotomous

Patients with a diagnosis of COPD will be identified as having the condition. The disease will be treated as a dichotomous variable, with the patient being flagged as either having or not having the condition.

PNEUMONIA

-Dataset Location= local UMHS MPOG database

-Variable Name= 70138 Respiratory - Bronchitis / Pneumonia

-Variable Type= Dichotomous

Patients with a diagnosis of pneumonia will be identified as having the condition. The disease will be treated as a dichotomous variable, with the patient being flagged as either having or not having the condition.

DIABETES

-Dataset Location= local UMHS MPOG database

-Variable Name= 70046 Endocrine - Diabetes

-Variable Type= Categorical

Patients with a diagnosis of diabetes will be identified as having the condition. The disease will be treated as a categorical variable, with the patient being flagged as either having or not having the condition by type.

LIVER DISEASE

-Dataset Location= local UMHS MPOG database

-Variable Name= 70052 GI - Liver Disease

-Variable Type= Dichotomous

Patients with a diagnosis of liver disease will be identified as having the condition. The disease will be treated as a dichotomous variable, with the patient being flagged as either having or not having the condition.

RENAL FAILURE

-Dataset Location= local UMHS MPOG database

-Variable Name= 70060 Renal / Urologic - Renal Failure

-Variable Type= Dichotomous

Patients with a diagnosis of renal failure will be identified as having the condition. The disease will be treated as a dichotomous variable, with the patient being flagged as either having or not having renal failure.

HISTORY OF CAD

-Dataset Location= local UMHS MPOG database

-Variable Name= 70027 Cardiovascular

-Variable Type= Dichotomous

Patients with a diagnosis of Coronary Artery Disease will be identified as having the condition. The disease will be treated as a dichotomous variable, with the patient being flagged as either having or not having a history of CAD.

RECENT CAD EVENT

-Dataset Location= Centricity

-Variable Name= Cardiovascular>MI> either “less than 7 days” OR between 7 and 30 days”

-Variable Type= Dichotomous

Recent Myocardial Infarction is a well-known comorbidity affecting negative surgical outcomes.

CONGESTIVE HEART FAILURE

-Dataset Location= local UMHS MPOG database

-Variable Name= 70026 Cardiovascular - Congestive Heart Failure

-Variable Type= Dichotomous

Patients with a diagnosis of Congestive Heart Failure will be identified as having the condition. The disease will be treated as a dichotomous variable, with the patient being flagged as either having or not having the condition.

SENSORIUM OR COMA

-Dataset Location= Centricity

-Variable Name= Endo/Neuro/Pain>Neuro sign/Sx>acute altered mental status OR Chronic altered mental status OR Coma OR Delirium Tremens OR Encephalopathy

-Variable Type= Dichotomous

Patients with a diagnosis of coma will be identified as having the condition. Sensorium or coma will be treated as a dichotomous variable, with the patient being flagged as either having or not having the condition.

PRIOR NEUROLOGIC CONDITION

-Dataset Location= local UMHS MPOG database

-Variable Name=

70085 Neuro - Cerebral Aneurysm

70086 Neuro - Cerebrovascular Disease

70087 Neuro - CNS Tumor

70088 Neuro - Cerebrovascular Accident

70089 Neuro - Dementia

70090 Neuro - Other

70091 Neuro - Peripheral Nerve Disease

70093 Neuro - Seizures

70094 Neuro - Symptoms

-Variable Type= Dichotomous/Categorical

Patients with a diagnosis of a preexisting neurologic condition will be identified as having the condition. The disease will be treated as a dichotomous variable, with the patient being flagged as either having or not having a prior neurologic condition. Conditions listed under “other neurological conditions” include ALS, Alzheimer’s Disease, Cerebral Aneurysm, Cerebral Palsy, Cervical Radiculopathy, CNS tumor, CVA, dementia, developmental delay, Eclampsia, Encephalocele, H/O head trauma, increased ICP, lumbar radiculopathy, mental retardation, migraine headaches, multiple sclerosis, myelomeningocele, neuropathy, Parkinson’s Disease, PNS disorder, Restless Leg Syndrome, Spina Bifida, spinal cord injury, subarachnoid hemorrhage, subdural hemorrhage, Tethered Cord Syndrome, and TIA.

MYASTHENIA GRAVIS

-Dataset Location= local UMHS MPOG database

-Variable Name= Aims_preop, 70090

-Variable Type= Dichotomous

Patients with a diagnosis of Myasthenia Gravis will be identified as having the disease. The disease will be treated as a dichotomous variable, with the patient being flagged as either having or not having cancer.

CANCER

-Dataset Location= local UMHS MPOG database

-Variable Name=

70068 Hematologic - Malignancy

70100 Misc - Malignancy (Solid or Hematologic)

70101 Misc - Solid Organ Malignancy Metastasis

-Variable Type= Dichotomous

Patients with a diagnosis of cancer will be identified as having the disease. The disease will be treated as a dichotomous variable, with the patient being flagged as either having or not having cancer.

STEROID USE

-Dataset Location= local UMHS MPOG database

-Variable Name= 70076 General - Medications - Chronic Steroid Use

-Variable Type= Dichotomous

Chronic steroid use will be noted from the patients’ history and will be treated as a dichotomous variable.

PREOPERATIVE SEPSIS

-Dataset Location= local UMHS MPOG database

-Variable Name= 70039 ID - Sepsis

-Variable Type= Dichotomous

Patients with a diagnosis of sepsis will be identified as having the condition. The condition will be treated as a dichotomous variable, with the patient being flagged as either having or not having the condition.

PRIOR OPERATION WITHIN 30 DAYS

-Dataset Location= Centricity Anesthesia H&P

-Variable Name= Prior Operation

-Variable Type= Dichotomous

This variable will be manually calculated by our programmer. The data query looks at each individual patient identifier and looks for prior completed intraoperative anesthesia record. The variable will be coded in a binary fashion (yes/no). Patients with prior surgery within 30 days are excluded from the analyses.

AMERICAN SOCIETY OF ANESTHESIOLOGISTS (ASA) PHYSICAL STATUS CLASSIFICATION

-Dataset Location= local UMHS MPOG database

-Variable Name= ASA_Class

-Variable Type= Categorical

The ASA status is a mature risk assessment tool with 6 categorical values and has repeatedly been demonstrated to be one of the strongest perioperative risk adjustment variables available.

HOME MEDICATIONS

-Dataset Location= local UMHS MPOG database

-Variable Name= 70077 General-Medications-Current

-Variable Type= Categorical

All home medications are categorized according to the National Library of Medicine RxNorm categorization system, allowing facile retrieval and risk adjustment of underlying patient risk. Preoperative opioid intake was identified if the preoperative admission medication list contained any of the following: dilaudid, morphine, vicodin, methadone, percocet, hydrocodone, fentanyl, vicoprofen, norco, duragesic, oxycodone, roxicodone, fioricet, percodan, lortab, lorcet, codeine, opium, hydromorphone, oxycontin, suboxone, endocet, or buprenorphine.

**PROCEDURAL AND ANESTHETIC RISK FACTORS**

SURGICAL COMPLEXITY SCORE: The surgical complexity score was derived using a previously established technique from the primary CPT code.[[25](#_ENREF_25),[26](#_ENREF_26)] This approach computes a continuous score using a logistic regression model to predict the study primary outcome. Adjusted estimates for each CPT code were then converted to the logarithmic scale and applied to model as an independent variable.

PRIMARY ANESTHESIA CPT CODE (Exclude liver transplant, labor/delivery, caesarian section)

-Dataset Location= local UMHS MPOG database

-Variable Name= Charge_Capture_Primary_Anesthesia_Code

-Variable Type= Categorical

Procedure type will be identified for exclusion purposes. These anesthesia code types are an additional method to ensure the accuracy of data query in completely excluding cases deemed inadmissible for the analysis.

DURATION OF PROCEDURE (ANESTHESIA, IN-ROOM, AND SURGICAL)

-Dataset Location= local UMHS MPOG database

-Variable Name=

Anesthesia_Start_DT

Anesthesia_End_DT

Patient_In_Room_DT

Patient_Out_Of_Room_DT

Procedure_Start_DT

Procedure_End_DT

-Variable Type= Continuous

Duration of the procedure will be recorded as a continuous variable and used for analyses to determine any correlation between length of procedure and postoperative morbidity. We will also use the anesthesia time to determine dose tertiles of NMB use.

AMINOGLYCOSIDE GIVEN

-Dataset Location= local UMHS MPOG database

-Variable Name= Aims_intraopmedications, 10202 (gentamicin), 10203 (gentamicin), 10023 (Amikacin), 10131 (clindamycin), 10313 (neomycin), 10314 (neomycin), 10207 (neomycin)

-Variable Type= Dichotomous

Aminoglycoside administration will be reported as a dichotomous variable: yes/no for use of any of the listed aminoglycosides in the intraoperative medication administration record

USE OF GENERAL ANESTHESIA

-Dataset Location= local UMHS MPOG database /Centricity

-Variable Name= either of the two intubation variables

- Centricity Object_Sys 20442 __ mm __ ET tube taped at __ cm
- MPOG ID 50122 Intubation Endotracheal Tube Size

-Variable Type= Dichotomous

Use of general anesthesia will be identified for inclusion in this study. The variable will be reported as dichotomous: yes/no for use of general anesthesia.

USE OF NEURAXIAL ANESTHESIA

-Dataset Location= local UMHS MPOG database

-Variable Name= 50691 Categorized note - Neuraxial anesthesia

-Variable Type= Dichotomous

Use of neuraxial anesthesia will be reported as a dichotomous variable: yes/no for use of neuraxial anesthesia.

PERIPHERAL NERVE BLOCKADE

-Dataset Location= local UMHS MPOG database

-Variable Name= 50199 Monitoring - Neuromuscular blockade -- peripheral nerve stimulator placed

-Variable Type= Dichotomous

Use of a peripheral nerve blockade will be reported as a dichotomous variable: yes/no for use of peripheral nerve blockade.

Propofol INFUSION

- Variable(s) Used = (10377, 10378, 10453, 10572, 10577, 10578, 10579, 10597, 10639, 10649
- Dataset Location = local UMHS MPOG database
- Variable Type = dichotomous

Propofol use will be identified. The receipt of propofol will be treated as a dichotomous variable with “No Use of Propofol” serving as the reference group.

USE OF INHALED ANESTHETIC MEDICATION

-Dataset Location= local UMHS MPOG database

-Variable Name= Any of the following >0.5

3260 Isoflurane Exp%

3270 Sevoflurane Exp%

3280 Desflurane Exp%

-Variable Type= Dichotomous

Use of inhaled anesthetic medication will be treated as a dichotomous variable: yes/no if Isoflurane, Sevoflurane, or Desflurane are used in a quantity greater than 0.5. Each inhaled agent will be treated as a distinct dichotomous variable for logistic regression.

ANESTHETIC EXPOSURE

-Dataset Location= Centricity

-Variable Name=

3260 Isoflurane Exp%

3270 Sevoflurane Exp%

3280Desflurane Exp%

-Variable Type= Continuous

Typically, general anesthesia is maintained by continuous infusion of a modern inhaled anesthesia medication: isoflurane, sevoflurane, or desflurane. Every 60 seconds, the AIMS automatically records the concentration of the medication administered to the patient and expired by the patient. The depth of general anesthesia is calculated using a comparison of expired anesthetic concentration to the ED50 and ED95 for that medication, adjusted for patient age.

The following concepts will be evaluated for inclusion in the model

1. Median calculated depth of anesthesia adjusted for age: MAC (or expired agent) during anesthesia. Each 5 min period has a calculated MAC for the entire case MAC (age adjusted, with Propofol) = Propofol rate (in mcg/kg/min) / 150 + [ Expired Sevoflurane / 1.8 + + [ Expired Isoflurane / 1.17 + + [ Expired Desflurane / 6.6 + + [ Expired Nitrous / 104 ] x 10 ^ (.00269 x (age of patient – 40) ).[[24](#_ENREF_24)]
2. Highest age-adjusted MAC within 30 min before extubation. This value will be included as an independent continuous variable during sensitivity analyses to evaluate effect on outcome variance.

TOF VALUES AROUND EMERGENCE

-Dataset Location= local UMHS MPOG database

-Variable Name= Aims_intraopphysiologic, 3330

-Variable Type= Continuous

0,1 = deep, moderate (2), mild (3-4) measured within 30 minutes of extubation without subsequent dose NMB. Capture nil value as “no TOF measured.

NMB ADMINISTRATION

-Dataset Location= local UMHS MPOG database

-Variable Name=

cisatracurium MPOG ID 10129

vecuronium MPOG ID 10446

rocuronium MPOG ID 10393

Neostigmine (MPOG ID 10315)

Edrophonium (MPOG ID 10046, 10170)

Physostigmine (MPOG ID 10360)

-Variable Type=dichotomous for use of NMB, categorical for choice of drug

Neuromuscular blocking agents (NMBAs) and agents used for their reversal will be identified. NMBAs include any line item on the day of surgery indicating receipt of:, rocuronium, vecuronium, or cisatracurium. NMBA reversal agents include any line item on the day of surgery indicating receipt of: edrophonium, neostigmine or pyridostigmine.

To evaluate this risk, we will develop an ordinal categorical variable with the reference group being “No NMBA”, group 1 including “Any NMBA use without reversal agent use” and group 2 including “Any NMBA use with reversal agent use”.

NMB AMOUNT

-Dataset Location= local UMHS MPOG database

-Variable Name=

cisatracurium MPOG ID 10129

vecuronium MPOG ID 10446

rocuronium MPOG ID 10393

-Variable Type= continuous for dose, durations

Neuromuscular blocking agents (NMBAs) will be identified. NMBAs include any line item on the day of surgery indicating receipt of: suxamethonium or succinylcholine, rocuronium, vecuronium, atracurium, or cisatracurium. In each patient, we will calculate dose of NMB mg/kg IBW/hour of anesthesia time (continuous) for the relaxant that is used the greatest quantity throughout the procedure. Within each relaxant, the highest quartile of administered NMB/IBW/hr. anesthesia time will be converted to a dichotomous variable of high dose NMB.

INTRAOPERATIVE OPIOID DRUG/DOSE

- Variable(s) Used = Morphine MPOG ID 10306; Fentanyl MPOG ID 10186; Hydromorphone MPOG ID 10219, Naloxone MPOG ID 10312
- Dataset Location = local UMHS MPOG database
- Variable Type = dichotomous and continuous

Intraoperative (during the procedure) receipt of opiate pain medications will be identified. The opioids of interest are: Fentanyl, Sufentanil, Alfentanil, Morphine, Hydromophone Oxycodone, Remifentanil. The receipt of an intra-operative opiate use will be treated as a dichotomous variable with “No Use of intra-operative Opiate” serving as the reference group. Additionally, we will explore the use of the opioid antagonist naloxone. The receipt of an intra- operative naloxone use will be treated as a dichotomous variable with “No Use of intra-operative naloxone” serving as the reference group. The dose of opioid per mg/kg IBW/hour of anesthesia time (continuous variable) will be calculated for each patient. When multiple opioids are used total opioid equivalence will be calculated. Within each opioid, the highest quartile of administered opioid/IBW/hr. anesthesia time will be converted to a dichotomous variable of high dose opioid.
